# Inhibition of radiographic progression across levels of composite index-defined disease activity in patients with active psoriatic arthritis treated with intravenous golimumab: results from a phase-3, double-blind, placebo-controlled trial

**DOI:** 10.1186/s13075-020-2126-1

**Published:** 2020-03-06

**Authors:** Philip Mease, M. Elaine Husni, Shelly Kafka, Soumya D. Chakravarty, Diane D. Harrison, Kim Hung Lo, Stephen Xu, Elizabeth C. Hsia, Arthur Kavanaugh

**Affiliations:** 1 Seattle Rheumatology Associates, Swedish Medical Center/Providence St. Joseph Health and University of Washington School of Medicine, 601 Broadway, Suite 600, Seattle, WA 98122 USA; 2 Cleveland Clinic, Cleveland, OH USA; 3 Janssen Scientific Affairs, LLC, Horsham, PA USA; 4 Drexel University College of Medicine, Philadelphia, PA USA; 5 Janssen Research & Development, LLC, Spring House, PA USA; 6 University of Pennsylvania, Philadelphia, PA USA; 7 University of California, San Diego, San Diego, CA USA

**Keywords:** Psoriatic arthritis, Intravenous golimumab, Anti-TNF therapy, Biologic therapy, Radiographic progression, Composite indices

## Abstract

**Background:**

In the GO-VIBRANT trial of intravenous golimumab in psoriatic arthritis (PsA), golimumab significantly inhibited radiographic progression. In post hoc analyses, we evaluated changes in total PsA-modified Sharp/van der Heijde scores (SHS) across levels of composite index-defined disease activity following treatment.

**Methods:**

In this phase-3, double-blind, placebo-controlled trial, 480 bio-naïve patients with active PsA randomly received intravenous golimumab 2 mg/kg (*N* = 241; week 0, week 4, every 8 weeks [q8w]) or placebo (*N* = 239; week 0, week 4, week 12, week 20) followed by golimumab (week 24, week 28, q8w) through week 52. Week 24 and week 52 SHS changes in patient subgroups, defined by levels of disease activity as assessed by several composite measures (minimal disease activity [MDA], very low disease activity [VLDA], Psoriatic ArthritiS Disease Activity Score [PASDAS], Disease Activity in Psoriatic Arthritis [DAPsA], Clinical Disease Activity Index [CDAI]), were evaluated post hoc in 474 patients with evaluable radiographic data. Partially (last-observation-carried-forward methodology) and completely (nonresponder methodology) missing data were imputed.

**Results:**

Across indices, golimumab-treated patients demonstrated less radiographic progression than placebo-treated patients, regardless of disease activity state achieved via golimumab, from week 0 to 24 (e.g., mean changes in PsA-modified SHS were − 0.83 vs. 0.91, respectively, in patients achieving MDA and − 0.05 vs. 1.49, respectively, in those not achieving MDA). Treatment differences observed at week 24 persisted through week 52, despite placebo-randomized patients crossing over to golimumab at week 24 (e.g., mean changes in PsA-modified SHS from week 0 to 52 for golimumab- vs. placebo→golimumab-treated patients achieving MDA were − 1.16 vs. 1.19, respectively) and regardless of whether low disease activity was achieved (0.03 vs. 1.50, respectively, in those not achieving MDA). Consistent patterns were observed for disease activity assessed using VLDA, PASDAS, DAPsA, and CDAI composite endpoints.

**Conclusions:**

The extent of structural damage inhibition afforded by up to 1 year of intravenous golimumab treatment paralleled levels of PsA activity, with greater progression of structural damage observed in patients with sustained higher disease activity. Among patients not achieving low levels of disease activity across several composite indices, golimumab-randomized patients appeared to exhibit far less progression of structural damage than placebo-randomized PsA patients, illustrating a potential disconnect between responses, wherein golimumab can inhibit structural damage independent of clinical effect.

**Trial registration:**

ClinicalTrials.gov. NCT02181673. Registered 04 July 2014.

## Introduction

Psoriatic arthritis (PsA) is an immune-mediated inflammatory disorder characterized by a wide array of clinical manifestations, including axial and peripheral arthritis, psoriatic lesions, nail disease, enthesitis, dactylitis, impairment of physical function, and lower levels of reported health-related quality of life (HRQoL). The burden of PsA to the individual can be severe, with some patients developing destructive arthritis leading to bony erosion and loss of joint architecture. In an early PsA cohort, after 1–5 years, 47% of patients had ≥ 1 erosion, despite conventional treatment [1]. Bone erosions in patients with PsA, which appear to differ from those observed in rheumatoid arthritis (RA), can be quite extensive and contribute to significant joint deformity and disability [2].

Given that PsA manifestations may vary over time within individuals, and respond differently to therapy [3], and patients expect biologic agents to effectively treat all aspects of disease, physicians need to assess disease activity and patient overall response to include all aspects. Specifically, the Group for Research and Assessment of Psoriasis and Psoriatic Arthritis (GRAPPA) and Outcome Measures in Rheumatology (OMERACT) have concluded that remission of both disease- and patient-specific symptoms should be the ideal target, with low or very low disease activity being reasonable alternatives under certain circumstances [4, 5].

Composite scores, which combine different assessments into a single index, offer an efficient method for assessing disease activity in a disorder with heterogeneous manifestations such as PsA. The recently published American College of Rheumatology-National Psoriasis Foundation treatment recommendations for PsA highlight the difficulties in assessing efficacy in PsA with a single algorithm, owing not only to the high degree of heterogeneity in the presentation and course of PsA, but also to involvement of multiple domains in a single patient [3]. Not surprisingly, the GRAPPA-OMERACT group has not yet reached consensus on the use of a specific index, as existing indices have both advantages and disadvantages and require further validation [5]. Consensus was obtained, however, on the concept that composite indices should include musculoskeletal disease/peripheral arthritis, skin disease, and disease impact/HRQoL [5].

Golimumab (Janssen Biotech Inc., Horsham, PA, USA) is a fully human anti-tumor necrosis factor alpha (TNFα) monoclonal antibody shown to be effective in patients with RA, ankylosing spondylitis, and PsA [6]. Specific to PsA, sustained clinical efficacy and inhibition of structural damage was observed in patients with moderate-to-severe PsA in both the GO-REVEAL trial of subcutaneous (SC) golimumab [7, 8] and the GO-VIBRANT trial of intravenous (IV) golimumab [9, 10]. Given the importance of further evaluating the utility of composite indices to assess diverse manifestations in patients with PsA, we conducted post hoc analyses of data from GO-VIBRANT to assess changes in radiographic progression at 6 months and 1 year in patients with varying levels of composite index-defined disease activity following treatment during both the controlled and uncontrolled study periods.

## Methods

### Patients and study design

Details of the GO-VIBRANT study design and participant eligibility criteria have been reported [9, 10]. Briefly, eligible patients were bio-naïve, had PsA based on the ClASsification of Psoriatic ARthritis (CASPAR) criteria [11] for ≥ 6 months, and demonstrated active disease (swollen joint count [SJC] ≥ 5 and tender joint count [TJC] ≥ 5 at screening and baseline and screening C-reactive protein [CRP] ≥ 0.6 mg/dL), despite therapy with disease-modifying antirheumatic drugs (≥ 3 months) and/or nonsteroidal anti-inflammatory drugs (≥ 4 weeks) or an intolerance to these therapies. Participants also had active or a documented history of plaque psoriasis.

Enrolled patients were randomly assigned (1:1) to receive IV infusions of golimumab 2 mg/kg at weeks 0, 4, and every 8 weeks (q8w) thereafter or placebo at weeks 0, 4, 12, and 20. Stable doses of methotrexate (MTX; ≤ 25 mg/week) were permitted for patients receiving this treatment for ≥ 3 months prior to study start, and stable doses of nonsteroidal anti-inflammatory drugs and low-dose oral corticosteroids were permitted for patients receiving them for ≥ 2 weeks. Patients with < 5% improvement in SJC and TJC at week 16 could enter early escape, wherein specific changes in concomitant medications were permitted. At week 24, all patients in the placebo group crossed over to receive golimumab 2 mg/kg at week 24, week 28, and q8w thereafter. The final study agent infusion was at week 52.

### Clinical assessments and composite endpoints

The primary outcome measure was the American College of Rheumatology 20% improvement (ACR20) response criteria [12]. An independent joint assessor determined the TJC (*N* = 68) and SJC (*N* = 66). Patients assessed pain using a visual analog scale (VAS; 0–10 cm or 0–100 mm), and both patients and physicians assessed global disease activity with a VAS. Patients assessed physical function using the Health Assessment Questionnaire-Disability Index (HAQ-DI; score range, 0–3) [13]. Serum CRP concentrations were also determined (upper limit of normal, 0.287 mg/dL).

The independent joint assessor evaluated the presence (1) or absence (0) of enthesitis in each lateral elbow epicondyle, medial femoral condyle, and Achilles tendon insertion point using the PsA-specific Leeds Enthesitis Index (LEI; score range, 0–6) [14]. In each of 20 digits, the independent joint assessor assessed dactylitis as absent (0), mild (1), moderate (2), or severe (3), with a total score range of 0–60 [15, 16]. Among patients with ≥ 3% body surface area (BSA) involvement at baseline, psoriasis was assessed using the Psoriasis Area and Severity Index (PASI) [17].

Several composite measures of disease activity were employed in our analyses. Patients meeting five or more of the following seven criteria were determined to have minimal disease activity (MDA): TJC ≤ 1, SJC ≤ 1, PASI ≤ 1 among patients with BSA ≥ 3%, patient pain VAS ≤ 15 mm, patient global VAS ≤ 20 mm, HAQ-DI ≤ 0.5, and LEI ≤ 1. Patients meeting all seven criteria were deemed to have achieved very low disease activity (VLDA; evaluated post hoc) [18, 19]. Additional composite measures, determined post hoc employing component data available from preplanned study assessments, included the Psoriatic ArthritiS Disease Activity Score (PASDAS), the Disease Activity in Psoriatic Arthritis (DAPsA) score, and the Clinical Disease Activity Index (CDAI). The PASDAS [20, 21] was calculated using patient global disease activity VAS (arthritis and psoriasis), physician global disease activity VAS, 68-joint TJC, 66-joint SJC, CRP, enthesitis (LEI), dactylitis (scores of 0–3 recoded to 0–1, where any score > 0 equaled 1; range, 0–20) [22], and the physical component summary score of the 36-item Short Form Health Survey [23]. Disease activity cutoffs were remission (≤ 1.9), low (> 1.9–< 3.2), moderate (≥ 3.2–< 5.4), and high (≥ 5.4) [24]. The DAPsA score was calculated as the sum of the 68-joint TJC, 66-joint SJC, CRP, patient pain VAS, and patient global disease activity VAS [21]. The disease activity cutoffs were remission (≤ 4), low (> 4–≤ 14), moderate (> 14–≤ 28), and high (> 28) [25]. The CDAI (range, 0–76), a composite index validated to assess disease activity in RA patients and also employed in clinical practice to assess PsA disease activity, was calculated as the sum of the 28-joint TJC, 28-joint SJC, patient global disease activity VAS, and physician global disease activity VAS. The disease activity cutoffs were remission (≤ 2.8), low (> 2.8–≤ 10), moderate (> 10–≤ 22), and high (> 22) [26]. Note that MDA was the only one of these composite endpoints prespecified in the overarching clinical trial.

### Radiographic assessments

Radiographs of the hands (posteroanterior) and feet (anteroposterior) obtained at weeks 0, 24, and 52 (or at the time of early discontinuation) were centrally read by two independent and blinded readers [27] and scored using the total Sharp/van der Heijde score (SHS) with modifications for patients with PsA, i.e., inclusion of distal interphalangeal joints in the hands, as well as pencil-in-cup and gross osteolysis deformities [28, 29]. The total PsA-modified SHS (range, 0–528) sums the erosion (0–320) and joint space narrowing (JSN; 0–208) scores for 40 hand and 12 foot joints. Severity of JSN is scored as 0 (no JSN), 1 (asymmetrical or < 25% JSN), 2 (25 to < 50% JSN), 3 (50–99% JSN or subluxation), or 4 (absence of a joint space, presumptive evidence of ankylosis, or complete luxation) [29]. Higher SHSs and more positive change scores indicate more existing radiographic damage and more radiographic progression, respectively.

### Data analysis

In this post hoc analysis, we assessed changes in the total PsA-modified SHS from week 0 to week 24 and from week 0 to week 52 according to disease activity state achieved at week 24 and week 52, respectively, as assessed by the MDA, VLDA, PASDAS, DAPsA, and CDAI composite indices. Partially missing (last-observation-carried-forward methodology) and completely missing (nonresponder methodology) clinical efficacy data were imputed. Linear extrapolation was employed to impute missing SHS data. At week 24, treatment group comparisons within disease activity state subgroups employed analysis of variance on the van der Waerden-normal scores with no adjustment for multiplicity of testing. Thus, reported *p* values are descriptive in nature.

## Results

### Patient disposition and baseline characteristics

Patient disposition through week 24 [9] and week 52 [10] of GO-VIBRANT has been reported. Briefly, data were collected from September 2014 to March 2017 at 90 sites in 11 European and North American countries. In total, 480 patients contributed data to the efficacy analyses, including 239 in the placebo group and 241 in the golimumab group. Of these, 474 patients contributed data to structural damage analyses, including 237 in each of the placebo and golimumab groups [27]. Demographic and disease characteristics were generally well-balanced between the treatment groups, including baseline radiographic findings and disease activity. Approximately one half of patients had dactylitis, two thirds had enthesitis, and more than 80% had ≥ 3% BSA psoriasis skin involvement at baseline. Use of MTX (mean dose, 15 mg/week) and oral corticosteroids (mean dose, 7.5 mg/day) was reported by 70% and 28% of patients, respectively, at baseline (Table 1).

**Table 1:** Baseline patient and disease characteristics

	IV placebo	IV golimumab, 2 mg/kg	All patients
Number of patients	239	241	480
Age (years), mean (SD)	46.7 (12.5)	45.7 (11.3)	46.2 (11.9)
Male, *n* (%)	121 (50.6)	128 (53.1)	249 (51.9)
White, *n* (%)	237 (99.2)	241 (100)	478 (99.6)
Body mass index (kg/m^2^), mean (SD)	28.9 (6.2)	28.9 (6.4)	28.9 (6.3)
Duration of PsA (years), mean (SD)	5.3 (5.9)	6.2 (6.0)	5.8 (6.0)
Swollen joint count (0–66), mean (SD)	14.1 (8.2)	14.0 (8.4)	14.0 (8.3)
Tender joint count (0–68), mean (SD)	26.1 (14.4)	25.1 (13.8)	25.6 (14.1)
Patient pain VAS (0–10), mean (SD)	6.4 (2.1)	6.3 (2.1)	6.3 (2.1)
Patient global disease activity VAS (0–10), mean (SD)	6.3 (2.1)	6.5 (1.9)	6.4 (2.0)
Physician global disease activity VAS (0–10), mean (SD)	6.4 (1.6)	6.2 (1.7)	6.3 (1.6)
≥ 3% BSA psoriasis skin involvement, *n* (%)	198 (82.8)	196 (81.3)	394 (82.1)
PASI score (0–72), mean (SD)^1^	8.9 (9.0)	11.0 (9.9)	9.9 (9.5)
PASDAS, mean (SD)^2^	6.7 (1.1)	6.7 (1.1)	6.7 (1.1)
DAPsA, mean (SD)^3^	72.8 (32.1)	71.8 (34.0)	72.3 (33.0)
CDAI score (0–76), mean (SD)^2^	34.4 (13.1)	33.3 (12.5)	33.8 (12.8)
HAQ-DI (0–3), mean (SD	1.3 (0.6)	1.3 (0.6)	1.3 (0.6)
C-reactive protein (mg/dL), mean (SD)	2.0 (2.1)	1.9 (2.5)	2.0 (2.3)
Patients with dactylitis, *n* (%)	124 (51.9)	134 (55.6)	258 (53.8)
Dactylitis score (1–60)^4^, mean (SD)	9.9 (10.1)	9.3 (9.4)	9.6 (9.7)
Patients with enthesitis, *n* (%)	181 (75.7)	185 (76.8)	366 (76.3)
Leeds Enthesitis Index score (1–6)^4^, mean (SD)	3.2 (1.6)	3.0 (1.6)	3.1 (1.6)
Total PsA-modified SHS (0–528), mean (SD)	34.5 (53.5)	35.5 (55.2)	35.0 (54.3)
Baseline use of:			
Methotrexate, *n* (%)	173 (72.4)	163 (67.6)	336 (70.0)
Mean (SD) dose (mg/week)	14.9 (4.8)	14.8 (4.7)	14.8 (4.7)
Oral corticosteroids, *n* (%)	67 (28.0)	66 (27.4)	133 (27.7)
Mean (SD) dose (mg/day)	7.6 (2.5)	7.4 (2.6)	7.5 (2.6)

^1^*n* = 188, 189, 377

^2^*n* = 227, 232, 459

^3^*n* = 236, 237, 473

^4^Among patients with dactylitis/enthesitis at baseline

*BSA* body surface area, *CDAI* Clinical Disease Activity Index, *DAPsA* Disease Activity in Psoriatic Arthritis, *IV* intravenous, *HAQ-DI* Health Assessment Questionnaire-Disability Index, *PASDAS* Psoriatic ArthritiS Disease Activity Score, *PASI* Psoriasis Area and Severity Index, *PsA* psoriatic arthritis, *SD* standard deviation, *SHS* Sharp/van der Heijde score, *VAS* visual analog scale

### PsA-modified SHS through week 24 and week 52

Individual reader assessments of the change from baseline in the total PsA-modified SHS were generally consistent with each other. The intra-class correlation coefficients for baseline and week 52 scores were 0.84 and 0.82, respectively, and 0.54 for week 52 change scores.

**Fig. 1:**
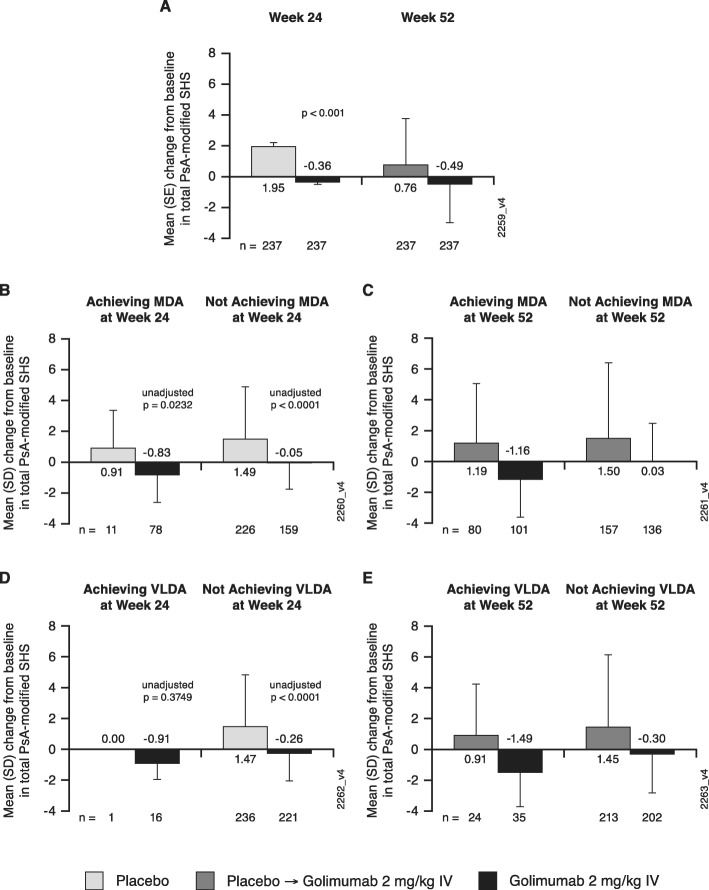
Mean changes from baseline in total PsA-modified SHS. Results are shown for all patients at week 24 and week 52 (**a**); patients who did and did not achieve MDA at week 24 (**b**) and week 52 (**c**); and patients who did and did not achieve VLDA at week 24 (**d**) and week 52 (**e**). IV, intravenous; MDA, minimal disease activity; PsA, psoriatic arthritis; SD, standard deviation; SE, standard error; SHS, Sharp/van der Heijde score; VLDA, very low disease activity

During the controlled period, mean changes from week 0 to week 24 in total PsA-modified SHS were − 0.36 in the IV golimumab group and 1.95 in the placebo group (*p* < 0.001). The greater inhibition of structural damage progression observed in the IV golimumab group at week 24 was sustained through week 52 (mean change in total PsA-modified SHS from week 0 to week 52, − 0.49). Patients randomized to placebo who crossed over to IV golimumab at week 24 (placebo→golimumab) exhibited a dampening of radiographic progression from week 24 to week 52 (mean change total PsA-modified SHS, − 0.64) relative to the period of placebo treatment (1.95), such that their overall mean change in SHS from week 0 to week 52 was 0.76 (Fig. 1a).

### Radiographic progression and disease activity assessed via composite indices

**Fig. 2:**
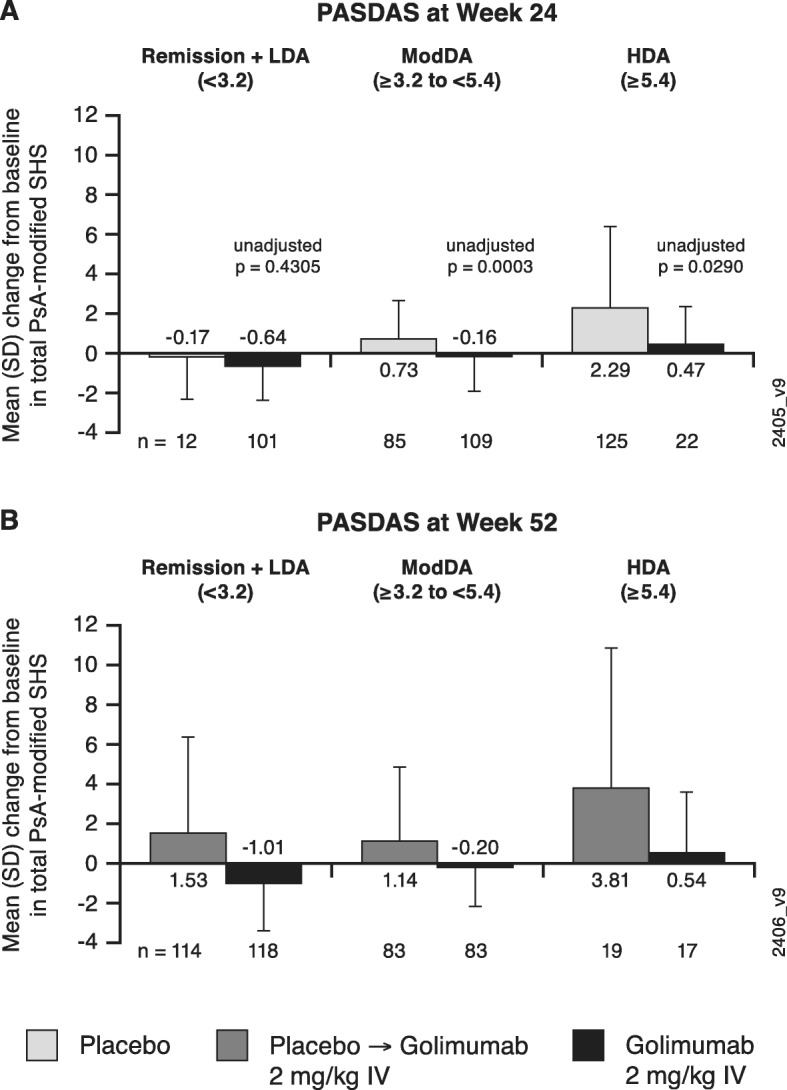
Mean changes from baseline in total PsA-modified SHS by PASDAS-defined disease activity state. Results are shown at week 24 (**a**) and week 52 (**b**). HDA, high disease activity; IV, intravenous; LDA, low disease activity; ModDA, moderate disease activity; PASDAS, Psoriatic ArthritiS Disease Activity Score; PsA, psoriatic arthritis; SD, standard deviation, SHS, Sharp/van der Heijde score

**Fig. 3:**
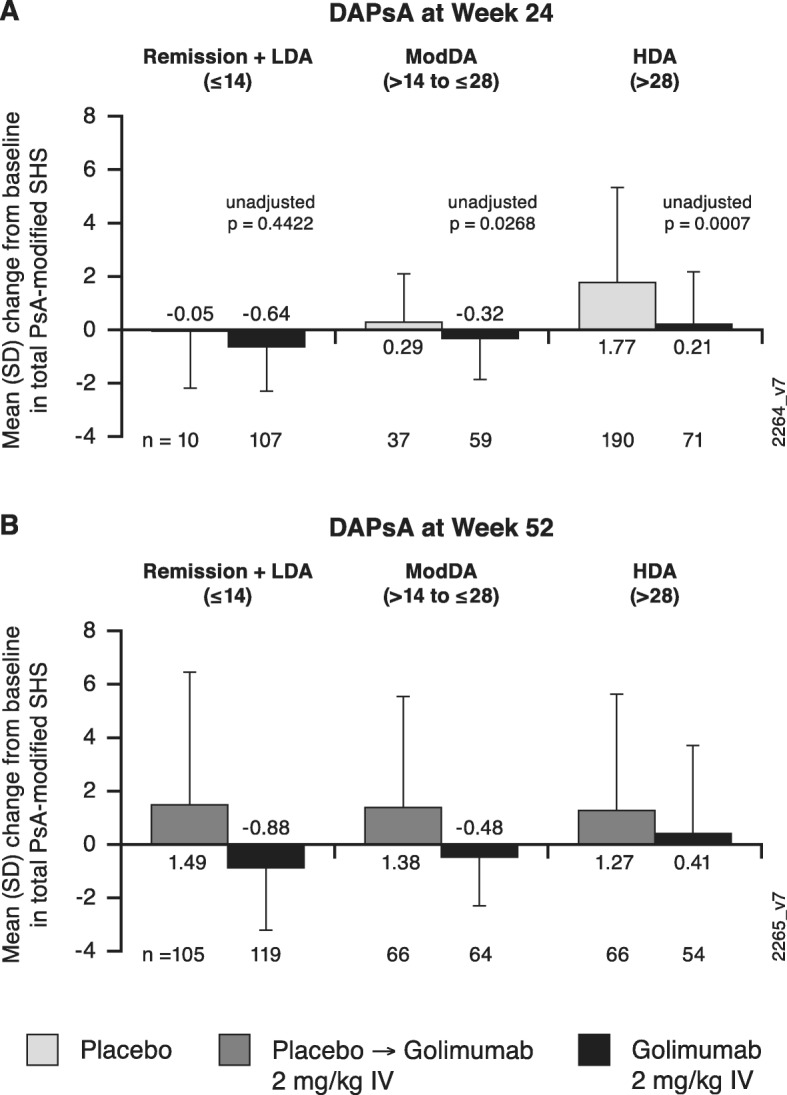
Mean changes from baseline in total PsA-modified SHS by DAPsA-defined disease activity state. Results are shown at week 24 (**a**) and week 52 (**b**). DAPsA, Disease Activity in Psoriatic Arthritis; HDA, high disease activity; IV, intravenous; LDA, low disease activity; ModDA, moderate disease activity; PsA, psoriatic arthritis; SD, standard deviation; SHS, Sharp/van der Heijde score

**Fig. 4:**
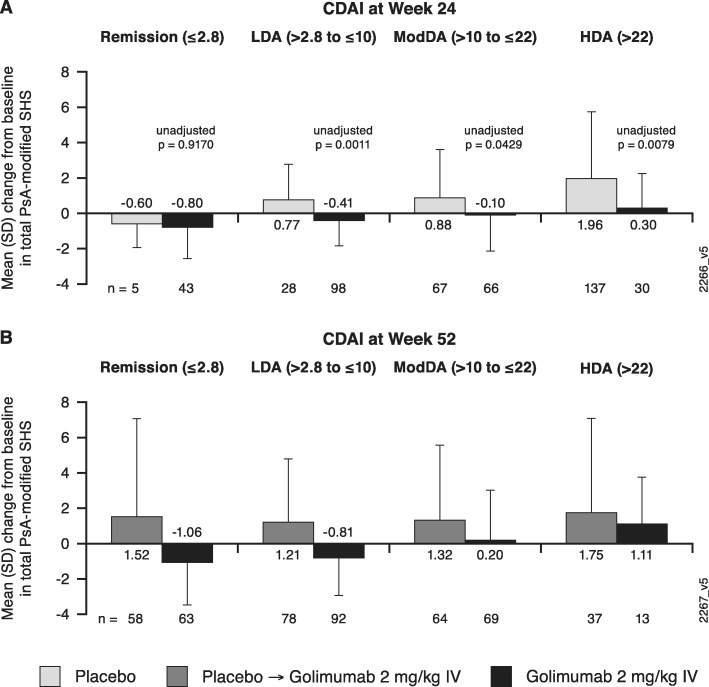
Mean changes from baseline in total PsA-modified SHS by CDAI-defined disease activity state. Results are shown at week 24 (**a**) and week 52 (**b**). CDAI, Clinical Disease Activity Index; HDA, high disease activity; IV, intravenous; LDA, low disease activity; ModDA, moderate disease activity; PsA, psoriatic arthritis; SD, standard deviation; SHS, – Sharp/van der Heijde score

Across composite indices, golimumab-treated patients demonstrated less radiographic progression than placebo-treated patients at week 24 within each category of disease activity (Figs. 1b, d; 2a; 3a; 4a). The observed treatment effect was sustained through week 52, i.e., numerically less radiographic progression was seen from week 0 to week 52 with golimumab than placebo→golimumab treatment regardless of composite index employed or disease activity state achieved (Figs. 1c, e; 2b; 3b; 4b).

When comparing the extent of radiographic progression across disease activity categories among patients receiving golimumab beginning at week 0, at both week 24 (Figs. 1b, d; 2a; 3a; 4a) and week 52 (Figs. 1c, e; 2b; 3b; 4b), individuals achieving lower levels of disease activity demonstrated numerically less disease progression than those whose disease activity remained high. For example, mean SHS changes in golimumab-randomized patients who achieved MDA or VLDA were − 0.83 and − 0.91, respectively, compared with − 0.05 and − 0.26 in patients who did not, at week 24 (Fig. 1b, d) and − 1.16 and − 1.49, compared with 0.03 and − 0.30 in patients who did not, at week 52 (Fig. 1c, e). Similarly, mean SHS changes from week 0 to week 52 were − 1.01, − 0.20, and 0.54 in golimumab-randomized patients demonstrating PASDAS-defined remission+low disease activity (LDA), moderate disease activity (ModDA), and high disease activity (HDA), respectively (Fig. 2b) and − 0.88, − 0.48, and 0.41 in patients demonstrating DAPsA-defined remission+LDA, ModDA, and HDA, respectively (Fig. 3b). Consistently, mean SHS changes from week 0 to week 52 were − 1.06 and − 0.81 in golimumab-randomized patients exhibiting CDAI-defined remission and LDA, respectively, compared with 0.20 and 1.11 in patients with CDAI-defined ModDA and HDA, respectively (Fig. 4b).

Of interest, patients receiving golimumab from week 0 to week 52 who did not achieve MDA or VLDA by week 52 still demonstrated numerically less radiographic progression than patients receiving placebo→golimumab, i.e., respective mean SHS changes from week 0 to week 52 were 0.03 vs 1.50 in MDA nonresponders and − 0.30 vs 1.45 in VLDA nonresponders (Fig. 1c, e). The observation of diminished radiographic progression in golimumab- vs. placebo→golimumab-treated patients despite still exhibiting HDA at week 52 was also observed for PASDAS (0.54 vs 3.81; Fig. 2b), DAPsA (0.41 vs 1.27; Fig. 3b), and CDAI (1.11 vs 1.75; Fig. 4b) composite indices.

## Discussion

The fully human anti-TNFα monoclonal antibody golimumab has demonstrated long-term clinical efficacy and inhibition of structural damage in patients with moderate-to-severe PsA [7–10]. Given the importance of assessing the diverse manifestations in patients with PsA [5], we conducted post hoc analyses to assess changes in radiographic progression in patients with varying levels of composite index-defined disease activity following treatment with IV golimumab or placebo in the large phase 3 GO-VIBRANT PsA trial [9, 10]. Composite endpoints were chosen for these analyses because they allow the assessment of multiple clinical manifestations in a single instrument [21]. Additionally, composite endpoints only require a single test of hypothesis and generally have no multiplicity issues, thereby potentially simplifying testing schemes in clinical trials [30]. As no consensus has been reached on the ideal composite endpoint [5], and each has advantages and limitations related to ease of use and domains included, we evaluated several indices, including the MDA, VLDA, PASDAS, DAPsA, and CDAI. Importantly, golimumab-treated patients exhibited less radiographic progression than placebo-treated patients, regardless of the composite index employed or extent of clinical disease activity attained. Further, the greater inhibition of structural damage progression observed with IV golimumab vs placebo at week 24 appeared to be sustained through week 52, despite placebo crossover to golimumab at week 24, indicating prompt treatment can have longstanding implications on structural progression.

Of interest, even in patients who did not achieve MDA/VLDA or who sustained high levels of disease activity assessed using the PASDAS, DAPsA, or CDAI indices, patients treated with golimumab IV exhibited less radiographic progression than those receiving placebo. This observation is consistent with other studies that have shown a disconnect between clinical and radiographic outcomes, suggesting a direct effect of golimumab on radiographic progression that is at least partially independent of its effect on clinical measures of disease activity. For example, evidence of an uncoupling of disease activity and radiographic progression was observed in adalimumab-treated PsA patients, whereby, irrespective of MTX use, inhibition of radiographic progression was greater than expected based on control of clinical disease activity alone [31]. Additionally, in RA patients treated via TNF inhibition, benefits in radiographic progression were observed among patients not achieving clinical improvement [32–34].

We hypothesize that these observations could relate to a direct effect of TNF inhibition on reducing osteoclast (OC) activity. Specifically, cultured peripheral blood mononuclear cells (PBMCs) obtained from patients with PsA have been shown to spontaneously release high quantities of biologically active TNFα [2], and in patients with PsA and other inflammatory arthritides, the PBMC pool is enriched with OC precursors (OCPs) [2, 35, 36]. Notably, increased levels of OCPs in PsA patients have been shown to be most pronounced in those with observable bone erosions, and blocking TNFα in such patients markedly decreased levels of circulating OCPs [2]. Further, in a small study of patients with RA or PsA, OCP populations correlated with peripheral blood TNFα levels [37]. Thus, observations to date suggest TNFα is pivotal in promoting OCP formation. The resulting OCs express receptors for macrophage colony-stimulating factor and receptor activator of nuclear factor-κB ligand (RANKL). RANKL, a member of the TNF family of cytokines, is the primary mediator of OC-induced bone resorption, as it is required for OC survival and activation through interaction with its receptor RANK [35, 36]. Thus, the observed synergy between TNFα and RANK in mediating OC-induced bone resorption [2, 38] suggests that the reduced OCP susceptibility to chemokine (e.g., TNFα) signals afforded by golimumab treatment could contribute to its direct control of structural disease progression. Indeed, in a previously reported study of PsA patients, SC golimumab had a beneficial impact on several biomarkers of bone remodeling, including monocyte/macrophage-derived chemokines [39].

The strengths of our analyses include a large patient population (*N* = 474) and moderate-to-good agreement in radiographic scores between independent central radiographic readers. Given the more variable occurrence and typically slower progression of structural damage generally observed in PsA than in other erosive diseases, as well the length of time required to fully assess radiographic outcomes, similar evaluations extending beyond 1 year would be useful to confirm our findings. Further, incorporation of biomarker determinations and assessments of new bone formations into future studies could enhance understanding the mechanism of a direct effect of golimumab on radiographic progression. While beyond the scope of the current post hoc analyses, prospective studies are needed to identify the composite endpoint(s) demonstrating strong correlation with inhibition of radiographic progression. As well, findings derived from patients enrolled into this randomized clinical trial may not be fully generalizable to a more heterogeneous PsA population.

## Conclusions

The extent of structural damage inhibition afforded by up to 1 year of IV golimumab treatment paralleled levels of PsA activity, with greater progression of structural damage observed in patients with sustained higher disease activity. Golimumab-randomized patients not achieving low levels of disease activity across a number of composite indices still demonstrated far less progression of structural damage than placebo-randomized PsA patients, suggesting a direct favorable effect of golimumab on radiographic progression at least partially independent of its effect on disease activity. Our findings provide additional evidence of a potential uncoupling of clinical and radiographic responses to TNF inhibition in patients with PsA.

## Data Availability

The datasets used and/or analyzed during the current study are available from the corresponding author on reasonable request.

## Abbreviations

ACR20: American College of Rheumatology 20% improvement
BSA: Body surface area
CASPAR: ClASsification of Psoriatic ARthritis
CDAI: Clinical Disease Activity Index
CRP: C-reactive protein
DAPsA: Disease Activity in Psoriatic Arthritis
GRAPPA: Group for Research and Assessment of Psoriasis and Psoriatic Arthritis
HAQ-DI: Health Assessment Questionnaire-Disability Index
HDA: High disease activity
HRQoL: Health-related quality of life
IV: Intravenous
JSN: Joint space narrowing
LDA: Low disease activity
LEI: Leeds Enthesitis Index
MDA: Minimal disease activity
ModDA: Moderate disease activity
MTX: Methotrexate
OC: Osteoclast
OCP: Osteoclast progenitor
OMERACT: Outcome Measures in Rheumatology
PASDAS: Psoriatic ArthritiS Disease Activity Score
PASI: Psoriasis Area and Severity Index
PsA: Psoriatic arthritis
RA: Rheumatoid arthritis
RANKL: Receptor activator of nuclear factor-κB ligand
SC: Subcutaneous
SD: standard deviation
SE: standard error
SHS: Sharp/van der Heijde score
SJC: Swollen joint count
TJC: Tender joint count
TNFα: Tumor necrosis factor alpha
VAS: Visual analog scale
VLDA: Very low disease activity
